# Assessment of hybrid machine learning algorithms using TRMM rainfall data for daily inflow forecasting in Três Marias Reservoir, eastern Brazil

**DOI:** 10.1016/j.heliyon.2023.e18819

**Published:** 2023-07-30

**Authors:** Ehab Gomaa, Bilel Zerouali, Salah Difi, Khaled A. El-Nagdy, Celso Augusto Guimarães Santos, Zaki Abda, Sherif S.M. Ghoneim, Nadjem Bailek, Richarde Marques da Silva, Jitendra Rajput, Enas Ali

**Affiliations:** a Department of Civil Engineering, College of Engineering, Taif University, P.O. Box 11099, Taif, 21944, Saudi Arabia; b Vegetal Chemistry-Water-Energy Research Laboratory, Faculty of Civil Engineering and Architecture, Department of Hydraulic, Hassiba Benbouali, University of Chlef, B.P. 78C, Ouled Fares, Chlef, 02180, Algeria; c Department of Civil Engineering, College of Engineering, Taif University, P.O. BOX 11099, Taif, 21944, Saudi Arabia; d Department of Civil and Environmental Engineering, Federal University of Paraíba, 58051-900, João Pessoa, Paraíba, Brazil; e Research Laboratory of Water Resources, Soil, And Environment, Department of Civil Engineering, Faculty of Civil Engineering and Architecture, Amar Telidji University, P.O. Box 37.G, 03000, Laghouat, Algeria; f Electrical Engineering Department, College of Engineering, Taif University, P.O. Box 11099, Taif, 21944, Saudi Arabia; g Laboratory of Mathematics Modeling and Applications, Department of Mathematics and Computer Science, Faculty of Sciences and Technology, Ahmed Draia University of Adrar, Adrar, 01000, Algeria; h Energies and Materials Research Laboratory, Faculty of Sciences and Technology, University of Tamanghasset, Tamanghasset, Algeria; i MEU Research Unit, Middle East University, Amman, Jordan; j Department of Geosciences, Federal University of Paraíba, 58051-900, João Pessoa, Paraíba, Brazil; k Water Technology Center, ICAR-IARI, New Delhi, 110012, India; l Faculty of Engineering and Technology, Future University in Egypt, New Cairo, 11835, Egypt

**Keywords:** Floods, Empirical mode decomposition (EMD), Brazilian river basin, Water resource management, Particle swarm optimization (PSO), Hydrological simulation

## Abstract

This study investigates the application of the Gaussian Radial Basis Function Neural Network (GRNN), Gaussian Process Regression (GPR), and Multilayer Perceptron Optimized by Particle Swarm Optimization (MLP-PSO) models in analyzing the relationship between rainfall and runoff and in predicting runoff discharge. These models utilize autoregressive input vectors based on daily-observed TRMM rainfall and TMR inflow data. The performance evaluation of each model is conducted using statistical measures to compare their effectiveness in capturing the complex relationships between input and output variables. The results consistently demonstrate that the MLP-PSO model outperforms the GRNN and GPR models, achieving the lowest root mean square error (RMSE) across multiple input combinations. Furthermore, the study explores the application of the Empirical Mode Decomposition-Hilbert-Huang Transform (EMD-HHT) in conjunction with the GPR and MLP-PSO models. This combination yields promising results in streamflow prediction, with the MLP-PSO-EMD model exhibiting superior accuracy compared to the GPR-EMD model. The incorporation of different components into the MLP-PSO-EMD model significantly improves its accuracy. Among the presented scenarios, Model M4, which incorporates the simplest components, emerges as the most favorable choice due to its lowest RMSE values. Comparisons with other models reported in the literature further underscore the effectiveness of the MLP-PSO-EMD model in streamflow prediction. This study offers valuable insights into the selection and performance of different models for rainfall-runoff analysis and prediction.

## Graphical abstract

**Figure:**
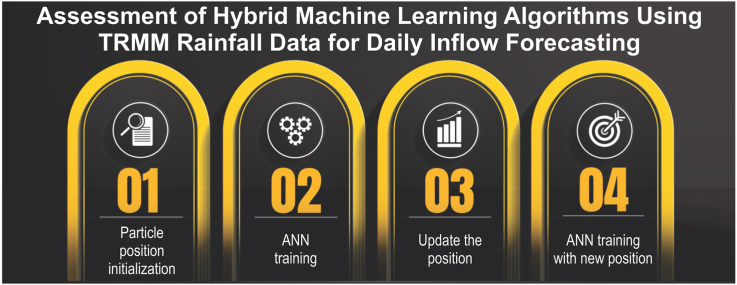

## 1 Introduction

Efficient water resource management has always played a crucial role in driving economic progress [1,2]. With growing water demand and challenges posed by prolonged droughts and climate uncertainties largely attributed to climate change, effective management of surface water reservoirs has become more important than ever. To tackle these challenges, it is essential to adopt effective reservoir management strategies. One promising approach is the utilization of hybrid machine learning algorithms, which can accurately predict future water demand, improve resource utilization efficiency, and minimize water waste. Accurate forecasting and estimation of surface flows and runoffs are essential for successful urban planning and the sustainable development of our communities. Thus, ensuring effective management of water resources is vital not only to meet the needs of present generations but also to ensure their availability for future generations.

The field of hydrology, along with other scientific disciplines, has experienced significant advancements since the inception of computer science in the 1960s. These advancements have led researchers to propose various hydrological models, specifically rainfall-runoff (*R*–R) models, which aim to transform rainfall processes into surface runoff [3,4]. Traditionally, the relationship between rainfall and runoff has relied on simplistic linear models based on empirical equations. However, these models fail to capture the complex reality of hydrological systems because of the high spatiotemporal variability of the data used. Consequently, estimates derived from these approaches often suffer from significant uncertainties and errors [5]. *R*–R models can be categorized differently based on their spatial and structural processing methods, including physical, empirical, conceptual, distributed, lumped, and semi-distributed models. Each category possesses distinct advantages and drawbacks [[6], [7], [8], [9]]. Moreover, climate change has complicated the rainfall-runoff relationship even further, leading to more nonlinear and intricate processes along with increased frequencies and severity of floods and droughts.

In recent years, machine learning algorithms have emerged as popular tools for prediction processes, particularly when dealing with limited information. These algorithms have proven valuable in assisting researchers and water resource managers in making informed decisions [[10], [11], [12], [13]]. For instance, a study by Ref. [14] utilized a blend of long short-term memory (LSTM) networks and bootstrap methods for daily river flow prediction, achieving reliable uncertainty estimation. In another study conducted in the Kohala and Garhihabibullah River basins in Pakistan, Ref. [15] compared extreme learning machines (ELM), LSTM networks, and random forest (RF) algorithms for monthly streamflow prediction. The results indicated that LSTM outperformed both ELM and RF methods, with ELM ranking second in terms of accuracy. Ref. [16] evaluated multiple machine learning models, such as artificial neural networks (ANN) with diverse training algorithms, RF, and locally weighted linear regression (LWLR), for streamflow estimation in the Oued Sebaou watershed in Northern Algeria. The analysis revealed that RF produced the best results in both the training and testing phases, while the ANN using the Levenberg-Marquardt algorithm achieved the second-ranking performance, surpassing both LWLR and other ANN variants. Additionally, Ref. [17] employed a convolutional neural network (CNN) to forecast monthly streamflow for the Huanren Reservoir and Xiangjiaba Hydropower Station in China. The study found that CNN consistently achieved superior performance compared to ANN and ELM regarding statistical metrics and stability. In the Shakkar watershed in the Narmada Basin, India, Ref. [18] explored the application of three artificial intelligence techniques - adaptive neuro-fuzzy inference system (ANFIS), Gaussian Process Regression (GPR), and ANN - for streamflow forecasting. The results demonstrated that ANFIS yielded the most accurate forecasts compared to GPR and ANN.

The Três Marias Reservoir (TMR), situated in the Upper São Francisco River basin, faces significant water scarcity challenges due to declining streamflow and increasing demand. Despite the basin's size and abundant natural resources, research on water resource management in this area continues to be a rich and ever-expanding field. Given the specific challenges faced by the TMR, this study aims to expand the range of flow prediction tools. We propose an approach that combines the Empirical Mode Decomposition-based Hilbert-Huang Transform (EMD-HHT) with Multilayer Perceptron (MLP) coupled with Particle Swarm Optimization (PSO), Gaussian Process Regression (GPR), and Generalized Regression Neural Network (GRNN). This integrated approach aims to simulate and forecast daily inflows into the TMR, facilitating improved water resource management in this region.

## 2 Related work

This section provides a comprehensive overview of pertinent research endeavors centered around the use of machine learning algorithms for streamflow and reservoir inflow prediction. Additionally, it encompasses studies that employed hybrid models, amalgamating machine learning techniques, empirical mode decomposition, and physics-based input features, to effectively forecast a variety of factors.

A noteworthy example of such hybrid modeling approaches can be found in the work of Ref. [19]. In their investigation, they presented a novel approach that integrates empirical mode decomposition (EMD), artificial neural networks (ANNs), and permutation entropy (PE) for wind speed forecasting. The EMD-PE-ANN model demonstrated superior predictive effectiveness compared to singular ANN models, achieving notable advancements in wind speed forecasting accuracy. This was substantiated by high correlation coefficients for short- and medium-term forecasts. Ref. [20] focused on load forecasting and presented a hybrid model that combines empirical mode decomposition (EMD) and neural networks (NN). Their approach demonstrated improved accuracy in multi-step ahead load forecasting compared to conventional methods, as indicated by the mean absolute percent error (MAPE). Ref. [21] utilized the Weather Research and Forecasting (WRF) model for rainfall prediction in tropical peatlands. They performed parameterization of the WRF for the maritime continent and identified the optimal combination of physical schemes for accurate rainfall prediction, contributing to the creation of a fire risk assessment system for Indonesian peatlands. Ref. [22] proposed a hybrid model that integrated an autoregressive integrated moving average (ARIMA) model with a random forest model for wind speed forecasting error prediction. Their study demonstrated that incorporating exogenous atmospheric variables, such as wind direction and temperature, improved the prediction of non-linear errors in wind speed forecasting. Ref. [23] addressed the prediction of China's Gross Domestic Product (GDP) using a novel grey forecasting model with a time power term. Their optimized grey model outperformed traditional grey models, providing more accurate forecasts of China's GDP, which can have significant implications for economic development and policy-making.

In the field of healthcare, a convolutional neural network (CNN) founded on the Hilbert-Huang Transform (HHT) and its derivatives were employed by Ref. [24] to forecast blood pressure risk levels utilizing photoplethysmography (PPG). Their model achieved high F1 scores in classification experiments, highlighting the effectiveness of the approach in predicting blood pressure status. Furthermore, several studies have focused on water resource management and hydrological modeling, particularly in predicting and simulating streamflow and reservoir inflows. Notably, Ref. [25] utilized the Multilayer Perceptron (MLP) and Support Vector Regression (SVR) in combination with the Improved Complete Ensemble Empirical Mode Decomposition with Additive Noise (ICEEMDAN) for monthly river flow forecasting in the Mangla watershed, Pakistan. The rainfall-runoff ICEEMDAN-(SVR) model yielded the best results with a root mean square error (RMSE) of 91.82, Nash-Sutcliffe efficiency coefficient (NSE) of 0.97, and correlation coefficient (R) of 0.97.

In another study, Ref. [26] adopted a joint approach that combined backpropagation artificial neural networks (BP-ANN) with Variational Mode Decomposition (VMD) and Ensemble Empirical Mode Decomposition (EMD) techniques to forecast the monthly streamflow of the Fentang reservoir in China. The VMD-BP model exhibited promising results in their study.

## 3 Study area and data sources

**Fig. 1:**
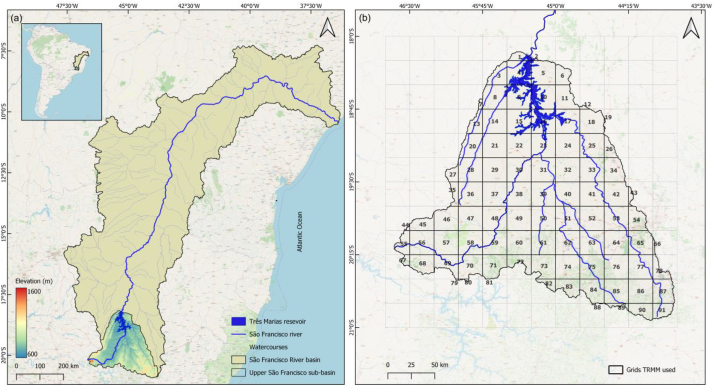
Geographic positioning of (a) Três Marias Reservoir in the Upper São Francisco River sub-basin, Eastern Brazil, and (b) the grid utilized for TRMM data retrieval and the respective areas of each measurement point within the Upper São Francisco sub-basin.

São Francisco River, spanning a length of 2914 km, holds great significance in Brazil. It stands as the longest river entirely within Brazilian territory and ranks 4th in South America and Brazil overall, following the Amazon, Paraná, and Madeira Rivers. Situated in the northeast-central part of Brazil, the river has a pivotal role in the country's development, underscoring the importance of comprehending its hydrological behavior for making effective water resource management decisions (Fig. 1a). The Upper São Francisco River basin spans between latitudes 18° 10′ S and 21° 10′ S and longitudes 43° 40′ W and 46° 40′ W (Fig. 1b) [27,28].

São Francisco River springs from the Canastra mountain range in the central-western region of Minas Gerais State, flowing northward through Minas Gerais and Bahia. It crosses the coastal range, draining a vast area exceeding 630,000 km^2^, before veering eastward to form the dividing line between Bahia (right bank) and the states of Pernambuco and Alagoas (left bank) [27,28]. Ultimately, it flows into the Atlantic Ocean. The region along the river's course is expansive and sparsely populated, with several cities, including Pirapora, São Francisco, Januária, and Bom Jesus da Lapa in Minas Gerais, and Petrolina, Juazeiro, and Paulo Afonso, emerging along its banks. The hinterland surrounding the river is characterized by an arid and sparsely inhabited nature, leading to the development of numerous small and isolated cities. Notably, Petrolina and Juazeiro have experienced significant growth and prosperity due to fruit production supported by irrigation [29].

### 3.1 TRMM-estimated rainfall data

**Fig. 2:**
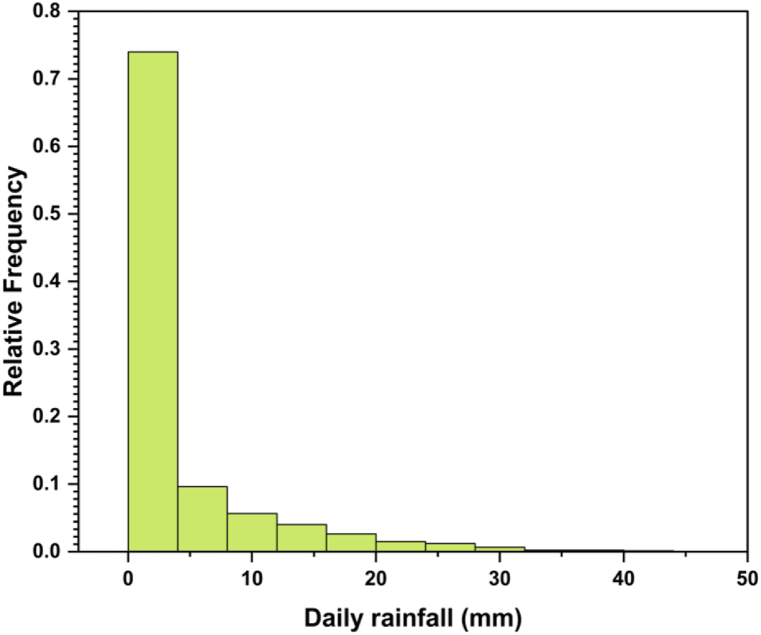
Distribution of daily rainfall amounts.

In this investigation, the data from the Tropical Rainfall Measuring Mission (TRMM) were utilized as a source of satellite-estimated rainfall information to examine the rainfall-runoff relationship at the TMR. Fig. 1b illustrates the grid utilized to download TRMM data over the Upper São Francisco sub-basin, and the proportional areas of each measurement point within the river basin. TRMM represents a collaborative effort between the Japanese space agency NASDA (now JAXA) and NASA, focusing on investigating tropical and subtropical precipitation — which makes up two-thirds of terrestrial precipitation — and modeling global climate processes [[30], [31], [32], [33]]. TRMM's primary objective is to establish a comprehensive database regarding rainfall distribution and latent heat exchanges in the region encompassing latitudes 35° N and 35° S. This area is predominantly occupied by oceans, leading to a dearth of surface and radiosonde atmospheric data. Similar to the inflow data, the TRMM rainfall series consists of 8036 values spanning from January 1, 1998, to December 31, 2019. Fig. 2 shows an analysis of daily rainfall (mm) and its relative frequency. The data reveal that a rainfall amount of 2 mm is the most frequent, accounting for approximately 73.99% of occurrences. As the rainfall amounts increase, the relative frequencies gradually decrease, indicating fewer occurrences. This trend continues for higher rainfall amounts, with relative frequencies decreasing for each subsequent increment.

The frequency distribution of daily rainfall, as depicted in Fig. 2, offers valuable insights into frequency patterns. Smaller rainfall amounts are more common, while larger amounts occur less frequently. This information is crucial for decision-making in various sectors, including agriculture, water resource management, and infrastructure planning, as it enables stakeholders to better understand the likelihood of different rainfall intensities in the area under analysis.

### 3.2 Streamflow data

**Fig. 3:**
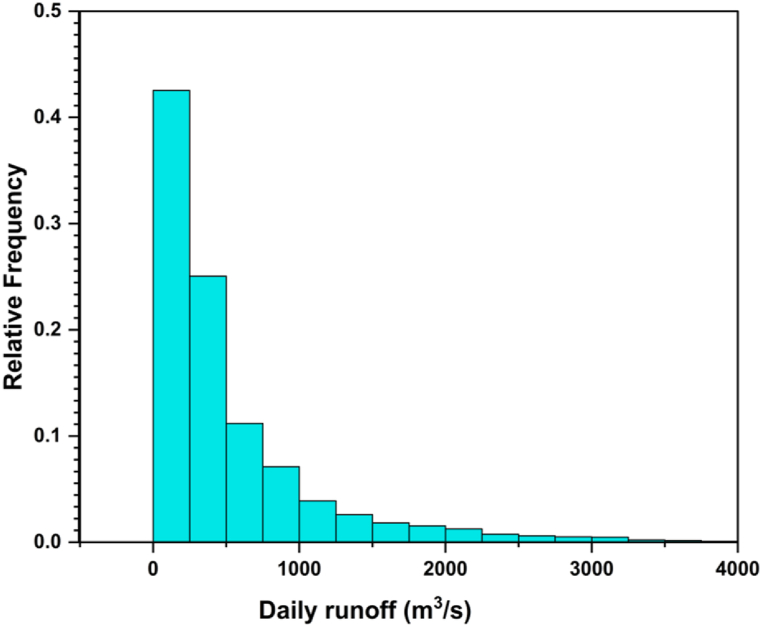
Daily runoff distribution in the analyzed area.

In our analysis of streamflow, we utilized the daily naturalized inflows to TMR as the time series. The naturalized inflow represents the volume of water that could have flowed into TMR without any alterations caused by storage, diversion, export, import, changes, or return flow in consumptive use, as stated in Ref. [32]. The Brazilian National System Operator (http://www.ons.org.br) provided the daily naturalized streamflow data (inflow data), which consists of 8036 values covering from January 1, 1998 to December 31, 2019. Fig. 3 depicts the distribution of daily runoff in the analyzed area. According to the data, the most common daily runoff value is 348 m³/s, with a relative frequency of approximately 46.52%. Moreover, a daily runoff value of 52 m³/s has a significant relative frequency, accounting for approximately 24.01% of occurrences, suggesting either a reverse flow or a decrease in water volume. As the runoff amounts increase, the relative frequencies gradually decrease. For instance, runoff values of 748 m³/s, 1148 m³/s, and 1548 m³/s have relative frequencies of approximately 14.35%, 5.99%, and 3.35%, respectively. This trend of decreasing relative frequencies continues for larger runoff amounts, such as 1948 m³/s, 2348 m³/s, 2748 m³/s, 3148 m³/s, and so forth. Consequently, the data indicates that moderate runoff levels are most common in the dataset.

Table 1 presents a summary of the statistical characteristics of the daily TRMM rainfall and Três Marias inflow data. The TRMM rainfall data show a maximum value of 52 mm, a minimum value of 0 mm, a mean of 3.67 mm, and a standard deviation of 6.73 mm. The coefficient of variation is 1.83, indicating moderate variability. The Três Marias inflow data exhibit a maximum of 4696 m³/s, a minimum of 0 m³/s, a mean of 524.8 m³/s, and a standard deviation of 609.7 m³/s. The coefficient of variation is 1.16, indicating relatively lower variability compared to the TRMM rainfall data. These statistical characteristics offer insights into the variability and distribution of the rainfall and inflow data, which are vital for further analysis and decision-making in water resource management.

**Table 1:** Statistical characteristics of daily TRMM rainfall and inflow used in the analysis.

Databases	Maximum	Minimum	Mean	STD	CV	Variance
TRMM (rainfall)	52 mm	0 mm	3.67 mm	6.73 mm	1.83	45.29 mm^2^
Três Marias (inflows)	4696 m³/s	0 m³/s	524.8 m³/s	609.7 m³/s	1.16	371,803 (m³/s)^2^

## 4 Gaussian Process Regression

Gaussian Process Regression (GPR) is a Bayesian non-linear technique widely employed in data-driven models for multi-output forecasting and database processing. Its popularity in artificial intelligence systems stems from its ease of implementation, provision of probabilistic significance output, and hyperparametric adaptive acquisition [13]. The key characteristics that define GPR are its reliance on the multivariable Gaussian Probability Distribution to model the stochastic process and its utilization of a linear combination of previous data observations to achieve unbiased forecasting [34].

Mathematically, GPR is defined by the mean function and the covariance-based kernel function. The input vectors *X*_*n*_ = (*x*_1_, *x*_2_, …, *x*_*n*_) and the output vectors *Y*_*n*_ = (*y*_1_, *y*_2_, …, *y*_*n*_) are assumed to be independently and identically distributed. The GPR equation is represented as follows:

(1) $$f\left(x\right)∼GP\left(m\left(x\right),k\;\left(x,x^{\prime }\right)\right)$$

where f(x) denotes the regression function. The Gaussian noise function for the observed *y* data points is given by:

(2) y=f(x)+ξ

(3) $$\xi =N\left(0,\sigma_{f}^{2}\right)$$

where ξ is the noise of the normal distribution function with zero mean and unit variance. According to Ref. [35], the noise combination in the covariance function *k* (*x*,*x*’) is described as follows:

(4) $$k\;\left(x,x^{\prime }\right)=\sigma_{f}^{2}\;\exp\left[\frac{-{\left(x,x^{\prime }\right)}^{2}}{2\tau^{2}}\right]+\sigma_{n}^{2}\sigma \left(x,x\right)$$

where σ(*x*,*x*) is the Kronecker delta function, and *n* represents the data points of *y*.

**Fig. 4:**
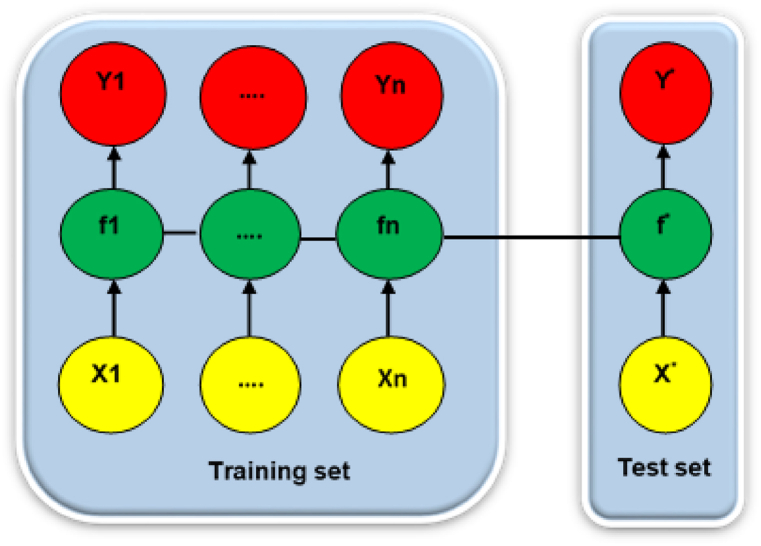
Flowchart of the GPR architecture.

According to Ref. [36], the four most important kernel functions for mapping the high-dimensional feature space of input series are presented in Table 2. Additionally, Fig. 4 illustrates a comprehensive flowchart of the GPR architecture, showing the sequential steps involved in the process and their interconnectedness. GPR enables the accurate and reliable representation of complex relationships between input and output variables, making it a valuable tool for analyzing the rainfall-runoff relationship at TMR.

**Table 2:** The four most commonly used kernel functions in GPR.

Kernel function	Equation
Poly kernel	$k\left(x_{i},x\right)={\left[\left(x_{i},x\right)+1\right]}^{d}$
Normalized poly kernel	$k\left(x_{i},x\right)=\frac{K\left(x_{i},x\right)}{\sqrt{K\left(x_{i},x_{i}\right)\;K\left(x,x\right)}}$
Radial basis kernel (RBF)	$K\left(x_{i},x\right)=e^{-\gamma {\left|x_{i}-x\right|}^{2}}$
Pearson VII kernel (PUK)	$k\left(x_{i},x\right)={\left[1+\left(\frac{2\sqrt{{\left\|x-x_{i}\right\|}^{2}}\;\sqrt{2^{-\omega }-1}}{\sigma }\right)\right]}^{-\omega }$

## 5 Generalized Regression Neural Network

Generalized Regression Neural Network (GRNN) is an algorithm created by Specht in 1991 [37]. It is designed to handle regression, prediction, and classification problems. GRNN stands out due to its highly parallel structure, which comprises a special linear and radial basis layer. It is a variant of radial basis neural networks and an enhanced version of traditional artificial neural networks (ANNs). The general equation of GRNN is given as follows:

(5) $$Y\left(x\right)=\frac{\sum_{k-1}^{N}y_{k}K\left(x,x_{k}\right)}{\sum_{k=1}^{N}K\left(x,x_{k}\right)}$$

In this equation, *Y*(*x*) represents the predicted or simulated value of the input *x*, *y*_*k*_ represents the activation weight of the pattern layer neuron at *k*, and *K* (*x*,*x*_*k*_) is the kernel function of the radial basic function kernel.

(6) $$K\left(x,x_{k}\right)=e^{\frac{{-d}_{k}}{{2\sigma }^{2}}}$$

where $d_{k}$ represents the squared Euclidean distance between the training samples $x_{k}$ and the input x:

(7) $$d_{k}={\left(x-x_{k}\right)}^{T}\;\left(x-x_{k}\right)$$

**Fig. 5:**
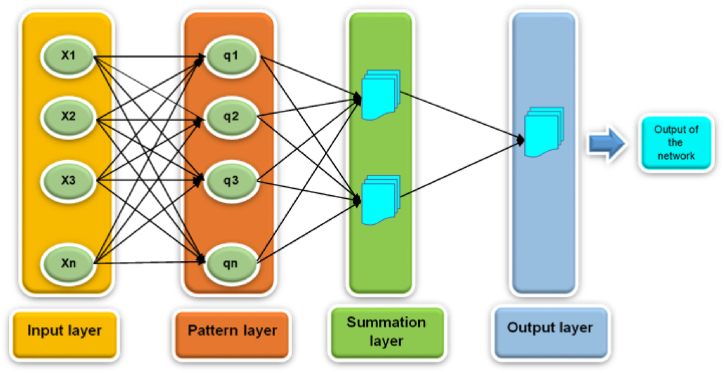
Flowchart of the GRNN architecture.

Fig. 5 provides a comprehensive flowchart depicting the GRNN architecture, illustrating the various steps and components involved in the process. By employing the GRNN architecture, we can achieve more accurate and reliable predictions of the complex connections between input and output variables in our study.

## 6 Multilayer Perceptron Optimized by Particle Swarm Optimization (MLP-PSO)

The Multilayer Perceptron (MLP) is a widely used artificial neural network consisting of multiple interconnected layers of neurons. It includes input, output, and hidden layers and is trained using backpropagation [6]. The input layer receives signals, the output layer performs tasks such as prediction [38], and the hidden layers serve as the primary computational engine for the MLP [39]. The output of a hidden neuron is determined by an activation function, the sum of weighted inputs, and a bias term (Eq. (8)):

(8) $$y=f_{act}\left(b+\sum_{i}\omega_{i}\;.\;x_{i}\right)$$

where $f_{act}$ represents the threshold from which a neuron will emit a signal, *x*_*i*_ denotes the signals arriving at the neuron, and ω_*i*_ refers to the weights that are adjusted throughout the learning process to enable efficient network prediction.

This study employed the Levenberg-Marquardt (LM) algorithm for the training and backpropagation of the Multilayer Perceptron (MLP). The LM algorithm was chosen due to its stability and effectiveness in finding solutions, making it widely preferred over other algorithms [[40], [41], [42]]. For instance, in a study conducted by Ref. [16], various backpropagation algorithms were compared for streamflow estimation, including Polak-Ribiére Conjugate Gradient, One Step Secant, Scaled Conjugate Gradient, Variable Learning Rate Backpropagation, and the LM algorithm. The comparison's results demonstrated that the MLP trained with the LM algorithm exhibited superior performance criteria compared to the other algorithms. The MLP used in this study utilized 50 neurons in the hidden layer. Though MLP is widely employed for classification and regression purposes, optimizing its weights and biases can be challenging.

Particle Swarm Optimization (PSO) is a stochastic optimization technique influenced by the collective behavior of bird flocking. It employs a group of candidate solutions, or particles, and iteratively improves them based on a predefined quality measure. PSO does this by moving particles within a search space using simple mathematical formulas based on their positions and velocities. The movement of each particle is affected by its locally most-favored position and directed towards superior positions in the search area [43,44], which get updated as improved positions are discovered.

**Fig. 6:**
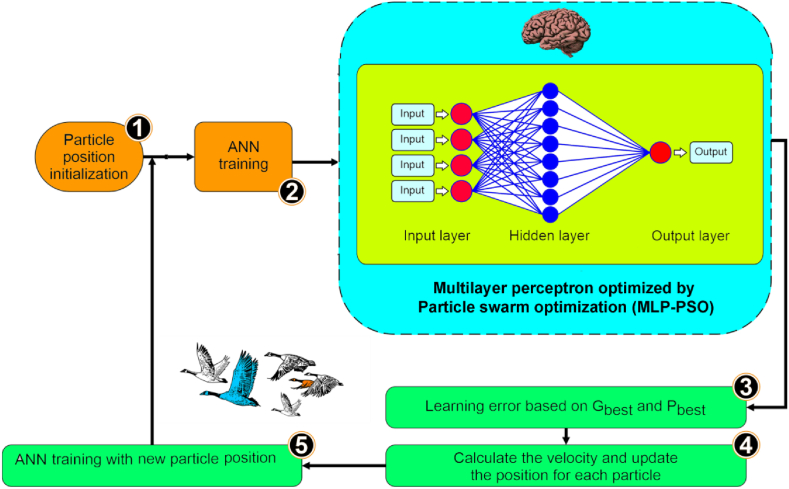
Flowchart of the MLP-PSO architecture.

By combining the strengths of the Multilayer Perceptron (MLP) architecture with the optimization capabilities of Particle Swarm Optimization (PSO), the MLP-PSO approach offers a novel solution for training neural networks and achieving optimal results. This approach has proven beneficial in a wide range of applications, including pattern recognition, time series prediction, and data classification, enhancing the MLP's capabilities and delivering superior results. Fig. 6 presents a comprehensive flowchart of the MLP-PSO architecture, illustrating the step-by-step process and the interconnectedness of each stage. This visual representation depicts how the MLP-PSO algorithm operates to improve the overall effectiveness and accuracy of the MLP.

In this study, the Particle Swarm Optimization (PSO) algorithm was employed with specific parameter settings outlined in Table 3. It was executed for a total of 1000 iterations using 100 swarms, guiding the search for optimal solutions. The variables were bounded within the range of −5 to 5, with υmax representing the lower bound and υmin the upper bound. The personal training coefficient (c1) was set to 1.5, and the global training coefficient (c2) was set to 2. A value of 1 was assigned to the inertia weight (w), and the inertia weight damping ratio (w_damp_) was set to 0.99. These carefully selected parameter values facilitated the effective exploration of optimal solutions within the study.

**Table 3:** Setting of the Particle Swarm Optimization (PSO) in this study.

Parameter	Value
Number of iterations	1000
Number of swarms	100
υ_max_ (lower bound of the variables)	5
υ_min_ (upper bound of the variables)	−5
c_1_ (personal training coefficient)	1.5
c_2_ (global training coefficient)	2
w (inertia weight))	1
w_damp_ (inertia weight damping ratio)	0.99

## 7 Empirical mode decomposition-based Hilbert Huang Transform

Hilbert-Huang Transform (HHT), founded on Empirical Mode Decomposition (EMD), is a prominent time-frequency analysis method first introduced in 1998 by Ref. [45] at NASA. Its primary objective was to facilitate the study and analysis of climate and earth science data by providing a robust time-scale approach for extracting and interpreting valuable information from complex databases and signals [46].

The HHT method consists of two main steps to effectively analyze non-stationary and non-linear signals. The initial step involves Empirical Mode Decomposition (EMD), which is employed to break down the initial signal into a limited set of Intrinsic Mode Functions (IMFs). Each IMF represents a single-component function characterized by a distinct frequency that varies over time. Notably, the first IMF captures the highest frequency component associated with each event present in the signal.

The subsequent step of the HHT is the application of the Hilbert Transform, which generates a pair of functions orthogonal to each IMF, exhibiting a phase shift of 90°. Mathematically, the signal x(t) can be represented as:

(9) $$x\left(t\right)=r_{n}\left(t\right)+\sum_{j=1}^{n}{IMF}_{j}\left(t\right)$$

where *x*(*t*) represents the time series, IMF_j_ is the *j*th oscillation, *r*(*t*) represents the residual of the decomposition, and *N* is the count of IMFs [47].

To ensure the accuracy and reliability of the decomposition into IMFs, specific admissibility conditions must be satisfied [48]. Firstly, each IMF must possess a zero mean. Secondly, the discrepancy between the quantity of extrema and zero crossings in an IMF should not exceed one. This condition guarantees that an IMF intersects the zero-axis between each minimum and maximum, thereby preventing unwanted fluctuations in the instantaneous frequency caused by signal asymmetry. Together, these conditions ensure the unique representation of the oscillatory mode within each IMF at any given time.

To obtain an IMF, the following stages are involved.

1. Employ cubic spline interpolation to create envelopes encompassing the minima and maxima points of the original signal, defining the minima envelope ($e_{\min}\;\left(t\right)$) and the maxima envelope ($e_{\max}\left(t\right)$).
2. Utilize the local mean of the minima envelope and maxima envelope to construct the mean envelope (*m*_*j*_(*t*)) using the equation:

(10) $$m_{j}\left(t\right)=\frac{e_{\max}\left(t\right)+e_{\min}\;\left(t\right)}{2}$$

3. Subtract the mean envelope from the original signal, resulting in the difference signal ($h_{j}\left(t\right)$):

(11) $$h_{j}\left(t\right)=x\left(t\right)-m_{j}\left(t\right)$$

If the mean envelope ($m_{j}\left(t\right)$) equals zero, the signal ($h_{j}\left(t\right)$) qualifies as an IMF. However, if the mean envelope is non-zero, the iterative process of IMF calculation continues until the criteria are met. This iterative procedure ensures the accurate extraction of each IMF, ultimately yielding a comprehensive decomposition.

In the last three decades, the EMD-based Hilbert Huang Transform has been applied in numerous hydrological studies as a noise-assisted data analysis method. Its significant capacity to denoise input time series has been demonstrated to enhance the accuracy of most analysis and forecasting algorithms [46,[49], [50], [51]].

## 8 Performance criteria

In this study, the effectiveness of the presented algorithms is evaluated utilizing several statistical metrics derived from the training and testing phases. The performance criteria employed are the mean absolute error (MAE), root mean square error (RMSE), Nash-Sutcliffe coefficient (NSE), and correlation coefficient (R). These metrics are computed according to equations (12)–(15), as detailed in the referenced studies [[52], [53], [54], [55], [56], [57], [58], [59], [60], [61]].

(12) $$MAE=\frac{1}{N}\sum_{i=1}^{N}\left|\left(Q_{obs,i}\right)-\left(Q_{sim,i}\right)\right|,\left(0\leq MAE<+\infty \right)$$

(13) $$RMSE=\sqrt{\frac{1}{N}\sum_{i=1}^{N}{\left[\left(Q_{obs,i}\right)-\left(Q_{sim,i}\right)\right]}^{2}},\left(0\leq RMSE<+\infty \right)$$

(14) $$NSE=1-\frac{\sum_{i=1}^{N}{\left[\left(Q_{obs,i}\right)-\left(Q_{sim,i}\right)\right]}^{2}}{\sum_{i=1}^{N}{\left[Q_{obs,i}-\bar{Q_{obs}}\right]}^{2}},\left(-\infty <NSE\leq +1\right)$$

(15) $$R=\frac{\sum_{i=1}^{N}\left(Q_{obs,i}-\bar{Q_{obs}}\right)\;\left(Q_{sim,i}-\bar{Q_{sim}}\right)}{\sqrt{\sum_{i=1}^{N}{\left(Q_{obs,i}-\bar{Q_{obs}}\right)}^{2}\;\sum_{i=1}^{N}{\left(Q_{sim,i}-\bar{Q_{sim}}\right)}^{2}}},\left(-1\leq R\leq +1\right)$$

where *Q*_*obs*,*i*_ and *Q*_*sim*,*i*_ are the observed and simulated data, respectively; *N* denotes the size of the time series, and $\bar{Q_{obs}}$ and $\bar{Q_{sim}}$ are the means of the measured and simulated data, respectively.

The dataset used in this study comprises 8036 values spanning a period of 22 years. For training purposes, approximately 5625 values (equivalent to around 15 years) were utilized, accounting for 70% of the dataset. The remaining 2410 values (equivalent to around 7 years) were allocated for the testing phase, representing 30% of the dataset. The division method chosen, with 30% for validation and 70% for training, aligns with the commonly employed approach in current studies [5,39,[62], [63], [64]]. This division ratio strikes a balance between utilizing a sufficiently large dataset for training and ensuring a robust validation process to assess the performance of the algorithms accurately.

## 9 Results and discussion

In this particular section, it is presented the outcomes derived from implementing the Gaussian Radial Basis Function Neural Network (GRNN), Gaussian Process Regression (GPR), and Multilayer Perceptron Optimized by Particle Swarm Optimization (MLP-PSO) models. These algorithms have been employed to analyze the rainfall-runoff relationship and predict runoff discharge in our study area. The effectiveness of each algorithm is evaluated based on various statistical measures, and their effectiveness in capturing the complex connections between input and output variables is compared. Moreover, we delve into the strengths and limitations of each model, providing insights into their applicability in real-world scenarios.

**Fig. 7:**
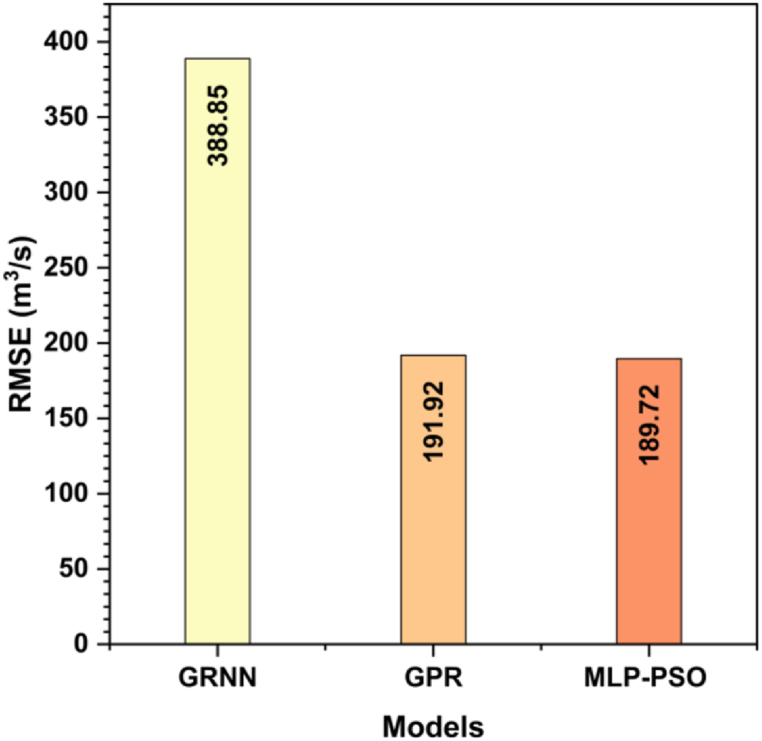
Comparison of RMSE during the training phase using the input combination of P_t+1_ and Q_t_.

Fig. 7 provides an initial visual assessment of the models' performance in accurately estimating the target variable. This figure illustrates the RMSE values for the GRNN, GPR, and MLP-PSO models when using the input combination of P_t+1_ and Q_t_ as input vectors. The GRNN model displays the highest RMSE of 388.849 m^3^/s, indicating a relatively larger deviation from the observed runoff discharge values. Conversely, the GPR model demonstrates improved performance with an RMSE of 191.923 m^3^/s, suggesting predictions closer to the actual values. However, it is worth noting that relying solely on the training phase for evaluating the models may not provide a comprehensive assessment of their performance. To ensure a robust evaluation, we also provide testing results using various input combinations. Table 4 presents these input combinations, labeled as Com.1 to Com.8, encompassing various combinations of input variables. The input vectors for each combination encompass P_t+1_ (one-day lagged precipitation), Q_t_ (current day discharge), Q_t−1_ (one-day lagged discharge), Q_t−2_ (two-day lagged discharge), and Q_t−3_ (three-day lagged discharge). The output variable, Q_t+1_, represents the discharge value for the following day. The use of these diverse input combinations allows us to explore the impact of different lagged variables and assess their influence on predicting future discharge accurately. Table 5 provides a detailed analysis of the RMSE comparison for the GRNN, GPR, and MLP-PSO models using different input combinations during the testing phase.

**Table 4:** Input combinations for GRNN, GPR, and MLP-PSO models.

Input combinations	Input vectors	Output
*Com.1*	*P*_*t*+1_ and *Q*_*t*_	*Q*_*t*+1_
*Com.2*	*P*_*t*+1_*, P*_*t*_, and *Q*_*t*_	*Q*_*t*+1_
*Com.3*	*P*_*t*+1_*, Q*_*t*_, and *Q*_*t*−1_	*Q*_*t*+1_
*Com.4*	*P*_*t*+1_*, P*_*t*_*, Q*_*t*_, and *Q*_*t*−1_	*Q*_*t*+1_
*Com.5*	*P*_*t*+1_*, Q*_*t*_*, Q*_*t*−1_, and *Q*_*t*−2_	*Q*_*t*+1_
*Com.6*	*P*_*t*+1_*, P*_*t*_*, Q*_*t*_*, Q*_*t*−1_, and *Q*_*t*−2_	*Q*_*t*+1_
*Com.7*	*P*_*t*+1_*, Q*_*t*_*, Q*_*t*−1_*, Q*_*t*−2_, and *Q*_*t*−3_	*Q*_*t*+1_
*Com.8*	*P*_*t*+1_*, P*_*t*_*, Q*_*t*_*, Q*_*t*−1_*, Q*_*t*−2_, and *Q*_*t*−3_	*Q*_*t*+1_

**Table 5:** RMSE comparison of models for different input combinations in the testing phase (in m^3^/s).

Input combinations	GRNN	GPR	MLP-PSO
Com.1	173.73	262.61	51.94
Com.2	166.30	208.00	52.78
Com.3	124.32	170.16	85.28
Com.4	124.86	134.31	56.66
Com.5	113.70	231.90	68.22
Com.6	116.29	192.86	63.79
Com.7	110.62	163.21	57.30
Com.8	114.15	74.57	57.33

The GRNN model exhibits varying RMSE values across different input combinations, ranging from 110.62 to 173.73 m^3^/s. The lowest RMSE is achieved with Com.7, while the highest is observed with Com.1. The GPR model also shows variability in its performance, with RMSE values ranging from 74.57 to 262.61 m^3^/s. Com.8 yields the lowest RMSE for GPR, while Com.1 has the highest. Similarly, the MLP-PSO model demonstrates variability in its RMSE values, ranging from 51.94 to 85.28 m^3^/s. Com.1 has the lowest RMSE for MLP-PSO, while Com.3 exhibits the highest value.

Comparing the models, it becomes evident that the MLP-PSO consistently outperforms the GRNN and GPR models across most input combinations. The MLP-PSO model achieves the lowest RMSE in Com.3, Com.4, Com.5, Com.6, and Com.7, indicating superior predictive accuracy in these cases. The GPR model shows competitive performance, achieving the lowest RMSE in Com.8. This capability allows the GPR model to efficiently deal with nonlinear patterns and capture complex dependencies that may exist in the precipitation flux relationship. However, it is noteworthy that the GRNN model generally has higher RMSE values compared to the other two models across all input combinations. A study conducted by other researchers [48], which compared the performance of GRNN and mixture-kernel GPR for seasonal streamflow forecasts in the Jinsha River catchment, is worth mentioning. The study found that GPR outperformed GRNN, especially in terms of deviation, degree of coincidence, and error level between predicted and observed values. These findings align with our analysis, where the GPR model consistently showed improved performance compared to the GRNN model.

**Fig. 8:**
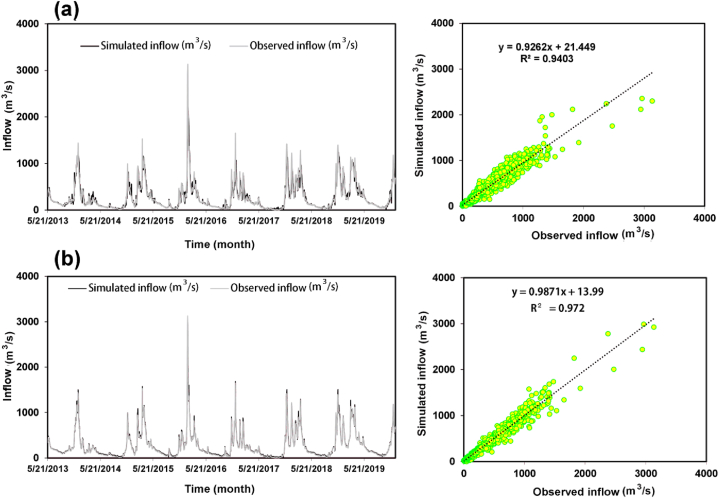
Comparison of observed and predicted daily inflow: (a) GPR model, (b) MLP-PSO model.

Taking into account the results from both Fig. 8 and Table 5, the MLP-PSO model stands out as the best-performing model overall. It consistently achieves the lowest RMSE values across different input combinations, demonstrating its effectiveness in capturing the complex rainfall-runoff relationship and accurately predicting runoff discharge. Compared to the other models, the MLP-PSO model shows significant improvement. For example, in Com.3, the MLP-PSO model achieves an RMSE of 85.28 m^3^/s, while the GRNN and GPR models have RMSE values of 124.32 and 170.16 m^3^/s, respectively. This represents a 31% improvement over GRNN and a 50% improvement over GPR. The superiority of the MLP-PSO model can be ascribed to its integration of the optimization capabilities of Particle Swarm Optimization (PSO) with the Multilayer Perceptron (MLP) architecture. The PSO algorithm effectively tunes the weights and biases of the MLP network, leading to improved prediction accuracy. The MLP-PSO model's consistent performance across multiple input combinations highlights its robustness and adaptability. For a more comprehensive view, we present the performance criteria (NSE, RMSE, MAE, and R) in Table 6.

**Table 6:** Performance criteria comparison for training and testing phases.

Phases	Models	MAE (m^3^/s)	RMSE (m^3^/s)	NSE	R
Training	GPR	32.900	55.703	0.993	0.997
	MLP-PSO	97.597	189.769	0.920	0.959
Testing	GPR	36.931	74.565	0.940	0.970
	MLP-PSO	27.918	51.947	0.971	0.986

In the training phase, the GPR model demonstrates robust performance, with a relatively low MAE of 32.9 m^3^/s and RMSE of 55.703 m^3^/s, indicating its ability to minimize average errors and limit the spread of errors from the observed values. The GPR model also exhibits a high NSE of 0.993 and a strong correlation coefficient (R) of 0.997, indicating excellent accuracy in capturing the variation and correlation between predicted and observed values during training. Conversely, the MLP-PSO model has higher MAE and RMSE values of 97.597 m^3^/s and 189.769 m^3^/s, respectively, indicating larger errors and a wider spread of errors compared to GPR. Despite this, the MLP-PSO model still exhibits decent performance with an NSE of 0.92 and an R of 0.959, indicating its capacity to capture the overall pattern and correlation between predicted and observed values, albeit with slightly higher errors compared to GPR.

In the testing phase, both models show a slight increase in errors compared to the training phase, an expected outcome when evaluated on unseen data. The GPR model maintains satisfactory performance with an MAE of 36.931 m^3^/s and RMSE of 74.565 m^3^/s, demonstrating its capacity to generalize well and provide reliable predictions on unseen data. It also upholds a relatively high NSE of 0.940 and an R of 0.970. In contrast, the MLP-PSO model demonstrates enhanced accuracy in the testing phase with an MAE of 27.918 m^3^/s and RMSE of 51.947 m^3^/s, indicating fewer errors and a narrower spread of errors compared to GPR. The MLP-PSO model achieves a higher NSE of 0.971 and a stronger R of 0.986, highlighting its superior performance in capturing the variation and correlation between predicted and observed values during testing.

Overall, the MLP-PSO model consistently outperforms the GPR model (Fig. 8a) with regard to MAE, RMSE, NSE, and R, during both the training and testing stages. The MLP-PSO model (Fig. 8b) exhibits an improvement of approximately 24% in MAE and 30% in RMSE in contrast to the GPR model, highlighting its superior accuracy and precision in predicting streamflow. The strong correlation between the model outputs and observed data, as depicted in Fig. 8, further validates the effectiveness of the chosen models for streamflow prediction.

In the next stage, we delve into the application of Empirical Mode Decomposition-Hilbert-Huang Transform (EMD-HHT) alongside the GPR and MLP-PSO models for streamflow prediction. EMD-HHT represents a data-driven method that decomposes the input time series into Intrinsic Mode Functions (IMFs) and extracts instantaneous frequency information using the Hilbert Transform.

**Fig. 9:**
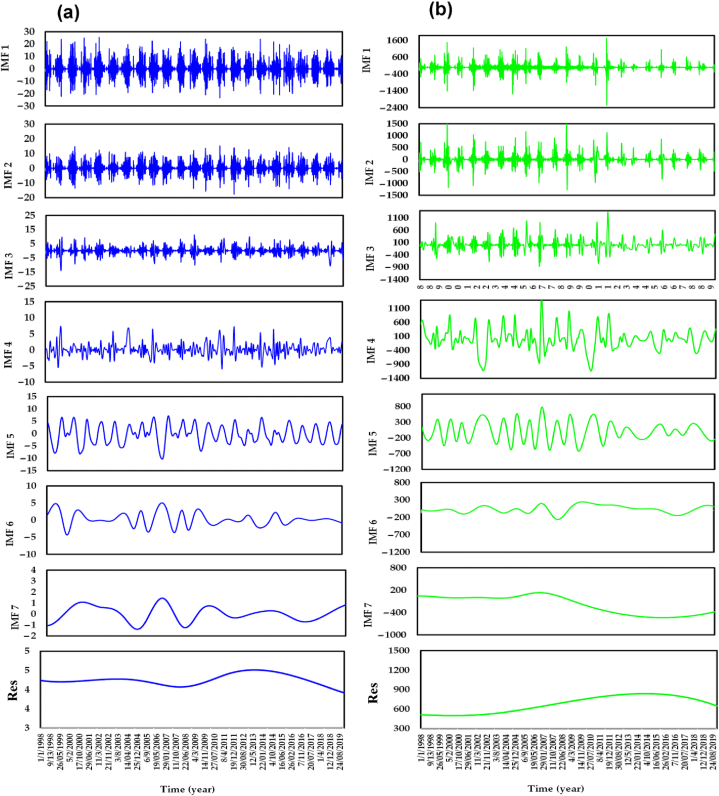
EMD daily time series of (a) TRMM rainfall and (b) inflows.

To implement EMD-HHT, the input data is decomposed into seven IMFs (IMF1, IMF2, IMF3, IMF4, IMF5, IMF6, and IMF7), along with a residual component (Res). The choice of seven components is based on achieving orthogonality in the decomposition results and minimizing the presence of spurious components, as depicted in Fig. 9a and b. It is critical to strike a balance in the number of components used for decomposition because too few components may not fully capture the underlying features of the raw data, while an excessive number of components can introduce computational challenges during model training. Previous studies have suggested that the center frequency aliasing phenomenon of the last component can be utilized to identify the most suitable number of decomposition modes. The performance criteria results for the top models, GPR-EMD and MLP-PSO-EMD, in the training and testing stages are given in Table 7. During the training phase, the GPR-EMD model demonstrates excellent performance with a low MAE of 22.648 m^3^/s and an RMSE of 34.984 m^3^/s. These values indicate the model's ability to minimize average errors and reduce the spread of errors from the observed streamflow values. The GPR-EMD model also exhibits a high NSE of 0.997 and a robust correlation coefficient (R) of 0.999, indicating its exceptional ability to capture the variation and correlation between predicted and observed values.

**Table 7:** Performance metrics of EMD-Enhanced models.

Phases	Models	MAE (m^3^/s)	RMSE (m^3^/s)	NSE	R
Training	GPR-EMD	22.648	34.984	0.997	0.999
	MLP-PSO-EMD	94.031	176.949	0.931	0.965
Testing	GPR-EMD	72.927	124.05	0.834	0.914
	MLP-PSO-EMD	32.507	60.032	0.961	0.982

Similarly, the MLP-PSO-EMD model exhibits strong performance in the training phase, with an MAE of 94.031 m^3^/s and an RMSE of 176.949 m^3^/s. Although these values are slightly higher than those of the GPR-EMD model, they still demonstrate the model's ability to capture the overall pattern and correlation between predicted and observed values. The MLP-PSO-EMD model achieves an NSE of 0.931 and an R of 0.965, demonstrating its ability to adequately capture the streamflow dynamics during training.

In the testing phase, both models experience a slight increase in errors compared to the training phase, as they are evaluated on unseen data. The GPR-EMD model exhibits a higher MAE of 72.927 m^3^/s and RMSE of 124.05 m^3^/s, indicating larger errors and a wider spread of errors compared to the training phase. However, the GPR-EMD model still maintains a reasonable level of performance with an NSE of 0.834 and an R of 0.914. On the other hand, the MLP-PSO-EMD model demonstrates improved accuracy in the testing phase with an MAE of 32.507 m^3^/s and RMSE of 60.032 m^3^/s. These values indicate smaller errors and a narrower spread of errors in contrast to the GPR-EMD model. The MLP-PSO-EMD model achieves a superior NSE of 0.961 and a stronger R of 0.982, highlighting its superior performance in capturing the streamflow dynamics during testing.

Overall, both the GPR-EMD and MLP-PSO-EMD models deliver promising results in streamflow prediction. The GPR-EMD model excels in terms of accuracy during the training stage, while the MLP-PSO-EMD model demonstrates superior performance in the testing stage. The MLP-PSO-EMD model showcases a significant percentage improvement of approximately 65% in MAE and 52% in RMSE in contrast to the GPR-EMD model during testing, indicating its effectiveness in capturing streamflow dynamics and making accurate predictions on unseen data.

Next, we assess the effect of incorporating various components on the accuracy of inflow prediction. Table 8 provides an overview of the different models and their corresponding decomposition schemes, using Empirical Mode Decomposition (EMD) with P_t+1_ and Q_t_.

**Table 8:** Variable combinations and outputs in EMD-based models.

Models	Decomposition by EMD *P*_*t*_+_1_ and *Q*_*t*_	Output
*M1*	*P*_*C*1_(*t* + 1), and *Q*_*C*1_(*t*)	*Q*_*t*_+_1_
*M2*	*P*_*C*1_(*t* + 1)*, Q*_*C*1_(*t*), and *Q*_*C2*_(*t*)	*Q*_*t*_+_1_
*M3*	*P*_*C*1_(*t* + 1)*, Q*_*C*1_(*t*)*, Q*_*C*2_(*t*), and *Q*_*C3*_(*t*)	*Q*_*t*_+_1_
*M4*	*P*_*C*1_(*t* + 1)*, Q*_*C*1_(*t*)*, Q*_*C*2_(*t*)*, Q*_*C*3_(*t)*, and *Q*_*R*_(*t*)	*Q*_*t*_+_1_
*M5*	*P*_*C*1_(*t* + 1)*, P*_*R*_ (*t* + 1), and *Q*_*C1*_(*t*)	*Q*_*t*_+_1_
*M6*	*P*_*C*1_(*t* + 1)*, P*_*R*_ (*t* + 1)*, Q*_*C*1_(*t)*, and *Q*_*C2*_(*t*)	*Q*_*t*_+_1_
*M7*	*P*_*C*1_(*t* + 1)*, P*_*R*_ (*t* + 1)*, Q*_*C*1_(*t*)*, Q*_*C*2_(*t*), and *Q*_*C3*_(*t*)	*Q*_*t*_+_1_
*M8*	*P*_*C*1_(*t* + 1)*, P*_*R*_ (*t* + 1)*, Q*_*C*1_(*t*)*, Q*_*C*2_(*t*)*, Q*_*C*3_(*t*), and *Q*_*R*_(*t*)	*Q*_*t*_+_1_

**Fig. 10:**
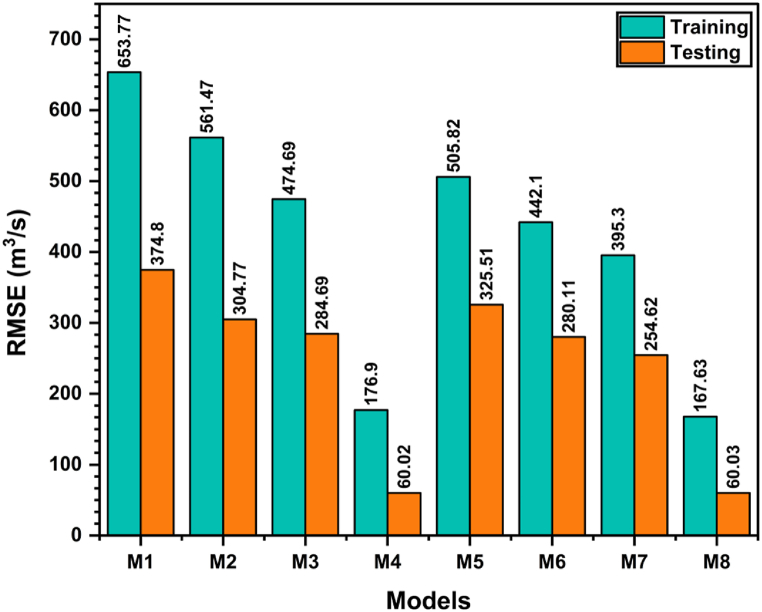
The RMSE values (m^3^/s) for MLP-PSO-EMD models in the testing phase.

Fig. 10 illustrates the RMSE values of the MLP-PSO-EMD model across different scenarios, denoted as M1, M2, M3, and so forth. The RMSE values offer valuable insights into the model's precision in both the training and testing stages. Upon analyzing the training RMSE values, it becomes apparent that as the models progress from M1 to M8, there is a noticeable reduction in the RMSE. Model M1 exhibits the highest training RMSE of 653.77, indicating larger errors in predicting the streamflow. However, as the model incorporates more components and variables, such as in M2, M3, and M4, the training RMSE decreases significantly. Notably, Model M4 records the lowest training RMSE of 176.90 m^3^/s, thereby indicating its superior ability to minimize errors and accurately model the observed streamflow during the training phase.

A similar trend is observed in the testing RMSE values. Model M1 displays the highest testing RMSE of 374.80 m^3^/s, indicating larger errors in predicting streamflow on unseen data. However, as the model incorporates additional components and variables, such as in M2, M3, and M4, the testing RMSE experiences a substantial decrease. Once again, Model M4 stands out by achieving the lowest testing RMSE of 60.02 m^3^/s, surpassing the effectiveness of the other models with regard to accuracy on unseen data.

**Fig. 11:**
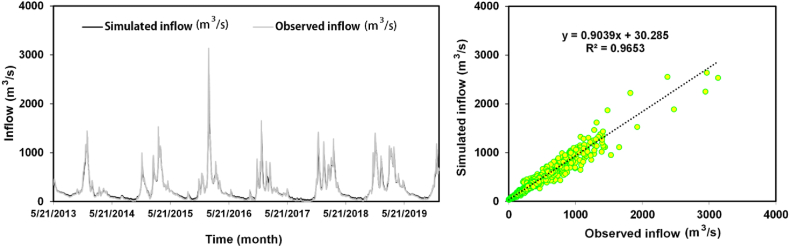
A comparison of observed and simulated daily inflow rainfall utilizing MLP-PSO-EMD.

Using the RMSE values as a basis, it becomes evident that the simplest and best-performing model among the presented options in Table 8 is Model M4. This model, which incorporates P_C1_(t + 1), Q_C1_(t), Q_C2_(t), Q_C3_(t), and Q_R_(t), consistently achieves the lowest RMSE values in both the training and testing stages. With a training RMSE of 176.90 m^3^/s and a testing RMSE of 60.02 m^3^/s, Model M4 demonstrates superior accuracy in predicting streamflow, outperforming the other models. The compatibility between the predicted and actual results of observed and simulated daily inflow rainfall can be observed in Fig. 11, further reinforcing the strong performance of Model M4. In terms of simplicity and performance, Model M4 emerges as the most favorable choice among the presented scenarios.

Despite these notable improvements, the proposed models still face challenges in accurately forecasting high and extreme inflows. To address this limitation, it is suggested to enhance the models by incorporating input variables related to weather factors or integrating empirical and/or physically-based models with machine learning approaches. Previous studies, such as Ref. [65], have shown promising results by integrating a parsimonious hydrological model (GR4J) with various machine learning models to improve streamflow forecasting. These hybrid models have shown enhancements in forecasting low, median, and high flows, particularly in reducing the bias of underestimating high flows. For rainfall-runoff modeling, Ref. [6] utilized GR4J, IHACRES, and MISD-based multi-conceptual-machine learning approaches, such as MLP-WOA (whale optimization algorithm) and SVR. The findings indicated that IHACRES-based MLP-WOA models enhanced the RMSE of the IHACRES model by 27% (8.49 m^3^/s).

Table 9 provides additional statistical measures for the observed data and predicted streamflow values using the MLP-PSO and MLP-PSO-EMD models in both the training and testing stages. Looking at the mean values, we observe that the predicted values from both models closely align with the mean of the observed values, indicating their ability to capture the overall trend. The minimum and maximum figures reflect the range of streamflow observations, and both models successfully encompass the full range of the observed data. In terms of the standard deviation (STD), the MLP-PSO model exhibits a slightly lower STD in the training stage compared to the MLP-PSO-EMD model, suggesting a slightly tighter distribution of predicted values around the mean. The coefficient of variation (CV) values indicate a moderate level of variability in both models' predictions compared to the observed data, indicating their ability to capture the inherent variability in streamflow.

**Table 9:** Summary of statistical measures comparing the observed streamflow data with the predicted values from the MLP-PSO and MLP-PSO-EMD models (in m^3^/s).

Phases	Data	Mean	Min.	Max.	STD	CV
Training	Observed	634.38	0.00	4696.00	674.18	1.06
	MLP-PSO	629.35	3.88	3678.81	647.14	1.03
	MLP-PSO-EMD	634.04	54.83	3618.07	653.46	1.03
Testing	Observed	269.11	0.00	3134.00	304.63	1.13
	MLP-PSO	279.63	0.06	2987.04	304.94	1.09
	MLP-PSO-EMD	273.54	30.85	2637.25	280.27	1.02

Overall, the statistical measures presented in Table 9 suggest that both the MLP-PSO and MLP-PSO-EMD models perform reasonably well in predicting streamflow values. However, based on the analysis of Table 9 and the previously discussed RMSE values, Model M4 (MLP-PSO-EMD) emerges as the best and simplest model. It exhibits superior accuracy and outperforms other models by minimizing errors and faithfully representing the observed streamflow. Nonetheless, there is still room for improvement, particularly in accurately predicting high and extreme inflows. Previous studies [[65], [66], [67], [68]] have shown promise in addressing this challenge by integrating additional input variables and combining different modeling approaches. Thus, it is recommended to explore these avenues for further enhancement.

In light of the promising outcomes demonstrated by the MLP-PSO-EMD model, our objective in the final stage is to conduct a comprehensive comparison of its performance against various techniques utilized in previous studies for streamflow forecasting. This comparative analysis, presented in Table 10, aims to highlight the effectiveness of the MLP-PSO-EMD model relative to other existing techniques. The MLP-PSO-EMD model achieved an RMSE of 60.03 m^3^/s and an NSE of 0.96 when utilizing TRMM data from 1998 to 2019 on a daily basis. Comparing these results to the other models, it can be observed that the MLP-PSO-EMD model exhibits strong performance in regard to both RMSE and NSE. The model outperforms the HEC-HMS and TRMM model by Parisouj et al. (2021) in terms of NSE, demonstrating a higher accuracy in streamflow prediction. Additionally, the MLP-PSO-EMD model outperforms the LSTM-DR2M model by Yeditha et al. [69] and the Tank-LSSVM model by Kwon et al. [70] in terms of RMSE, indicating a lower level of error in streamflow prediction.

**Table 10:** Comparison of the MLP-PSO-EMD model from the present study with other models reported in the literature.

Models	Reference	Data type	Location	RMSE (m^3^/s)	NSE	Data Amount
HEC-HMS	Parisouj et al. [71]	TRMM	Gorgan Dam (Iran)	–	0.43	2002–2007 (daily)
LSTM	Yeditha et al. [69]	CHIRPS	Mahanadi river basin (India)	208.27	0.88	2000 to 2018 (daily)
DR2M	Adane et al. [72]	TRMM 3B43v7	Awash River Basin (Ethiopia)	–	0.82	1998–2010 (daily)
Tank-LSSVM	Kwon et al. [70]	SM data	The Yongdam Catchment (Republic of Korea)	12.91	0.85	2007 and 2016 (daily)
HP11	Zanial et al. [73]	SDSM and TRMM rainfall	The hydropower plant (Malaysia)	12.4	–	1980–1986 (daily)
LSTM	Tang et al. [74]	IMERG data	The Shouxi River (China)	4.49	0.92	2014–2020 (hourly)
ANN	Rachidi et al. [75]	Terra Climate data	Essaouira coastal basin (Morocco)	15.32	0.81	1984–2021 (monthly)
SOM	Nascimento et al. [26]	TRMM	Três Marias Reservoir (Brazil)	–	0.82	1998 to 2019 (monthly)
MLP-PSO-EMD	Present Study	TRMM data	Três Marias Reservoir (Brazil)	60.03	0.96	1998 to 2019 (daily)

Moreover, the quantity of data used for model training and testing should be taken into consideration. The MLP-PSO-EMD model exhibits competitive performance over a relatively extended data period, encompassing 22 years from 1998 to 2019. This underscores the significance of having an ample amount of data for training machine learning models, as a longer time series enables the model to capture diverse patterns and enhance its accuracy. Additionally, it is essential to acknowledge that the unique characteristics of the study area and dataset can influence the performance of different models. This highlights the need for a context-specific analysis.

## 10 Conclusions

This study aimed to analyze the rainfall-runoff relationship and predict the runoff discharge into the TMR, in the Upper São Francisco River basin, using Gaussian Radial Basis Function Neural Network (GRNN), Gaussian Process Regression (GPR), and Multilayer Perceptron Optimized by Particle Swarm Optimization (MLP-PSO) models. The effectiveness of each model was assessed based on various statistical metrics, and their strengths and limitations were discussed.

The outcomes indicated that the MLP-PSO model consistently outperformed the GRNN and GPR models across most input combinations. It achieved the lowest RMSE in several cases, indicating its superior predictive accuracy. The GPR model also exhibited competitive performance, particularly in capturing nonlinear patterns and complex dependencies in the precipitation flux relationship. However, the GRNN model generally had higher RMSE values compared to the other models for all input combinations, consistent with previous research findings.

The MLP-PSO model emerged as the best-performing model, consistently achieving the lowest RMSE values across different input combinations. It demonstrated significant improvement over the GRNN and GPR models, highlighting its effectiveness in capturing the complex rainfall-runoff relationship and accurately predicting runoff discharge. The integration of Particle Swarm Optimization (PSO) with the Multilayer Perceptron (MLP) architecture in the MLP-PSO model played a crucial role in enhancing its prediction accuracy.

Further analysis explored the utilization of Empirical Mode Decomposition-Hilbert-Huang Transform (EMD-HHT) in conjunction with the GPR and MLP-PSO models for streamflow prediction. Both the GPR-EMD and MLP-PSO-EMD models showed promising results, with the GPR-EMD model excelling in accuracy during the training stage and the MLP-PSO-EMD model demonstrating better performance in the testing stage. The MLP-PSO-EMD model exhibited significant improvement in MAE and RMSE in contrast to the GPR-EMD model during testing, indicating its effectiveness in capturing streamflow dynamics and making accurate predictions on unseen data.

Moreover, the study examined the impact of incorporating different components on the prediction accuracy of streamflow. The results revealed that Model M4, which incorporated specific components and variables, consistently achieved the lowest RMSE values in both the training and testing stages. This model demonstrated superior accuracy in predicting streamflow and emerged as the most favorable choice among the presented scenarios.

Comparisons with other models reported in the literature highlighted the competitive performance of the MLP-PSO-EMD model in streamflow prediction. It outperformed various models in terms of RMSE and NSE, showcasing its effectiveness in capturing the observed streamflow dynamics.

Overall, this undertaken study contributes to the understanding of rainfall-runoff relationships and provides valuable insights into the performance of different models for streamflow prediction. The MLP-PSO model, particularly when combined with EMD-HHT, emerges as a robust and accurate approach for streamflow prediction, with potential applications in water resource management, flood forecasting, and hydrological modeling. However, further research is necessary to investigate the performance of these models on diverse datasets and under varying hydrological conditions.

## Author contribution statement

Ehab Gomaa, Bilel Zerouali, Salah Difi, Khaled A. El-Nagdy, and Celso Augusto Guimarães Santos conceived and designed the experiments; performed the experiments; analyzed and interpreted the data; contributed reagents, materials, analysis tools or data; and wrote the paper. Zaki Abda, Sherif S. M. Ghoneim, Nadjem Bailek, Richarde Marques da Silva, Jitendra Rajput, and Enas Ali analyzed and interpreted the data and wrote the paper.

## Data availability statement

Data will be made available on request.

## Funding

The authors would like to acknowledge the Deanship of Scientific Research at 10.13039/501100006261Taif University for funding this workhe authors appreciate the support from. Additional support for this work was provided by 10.13039/501100003593Brazil’s National Council for Scientific and Technological Development (Grant No. 313358/2021-4 and 309330/2021-1). This study was also financed in part by the Brazilian Agency for the Improvement of Higher Education (Coordenação de Aperfeiçoamento de Pessoal de Nível Superior - CAPES) – Fund Code 001, and Universidade Federal da Paraíba, Brazil.

## Institutional review board statement

Not applicable.

## Informed consent statement

Not applicable.

## Supplementary materials

Not applicable.

## Declaration of competing interest

The authors declare that they have no known competing financial interests or personal relationships that could have appeared to influence the work reported in this paper.
